# Commentary: Dietary Polyphenols Promote Growth of the Gut Bacterium *Akkermansia muciniphila* and Attenuate High-Fat Diet-Induced Metabolic Syndrome

**DOI:** 10.3389/fimmu.2017.00850

**Published:** 2017-07-27

**Authors:** Blessing O. Anonye

**Affiliations:** ^1^Microbiology and Infection Unit, Division of Biomedical Sciences, Warwick Medical School, University of Warwick, Coventry, United Kingdom

**Keywords:** gut microbiota, polyphenols, *Akkermansia*, diet, obesity

Dietary polyphenols exert a range of beneficial outcomes on the intestinal microbiota, and in metabolic syndrome, such as anti-inflammatory, antioxidant, anticarcinogenic, and antidiabetic effects. Research by Roopchand and colleagues demonstrated that concord grape polyphenols (GP) led to changes in the gut microbiota and reduction in conditions associated with metabolic syndrome arising from high-fat diet (HFD) in mice (1). Mice were divided into three groups and fed with HFD only or HFD supplemented with 10% soy-protein isolate (SPI) or HFD supplemented with 10% GP-SPI, respectively, for 13 weeks. When compared to the other diet groups, mice on the GP-SPI diet had lower body weight and adiposity though the food intake was similar across the groups (1).

Also, mice on the GP-SPI diet reduced markers of systemic inflammation as IL-6 was undetectable, and low levels of TNF-α and bacterial lipopolysaccharide was detected in the serum in comparison to the SPI group. However, levels of cholesterol, triglycerides, and IL-1β in the serum were not significantly different from mice fed with the SPI diet (1).

So, how did the GP-based diet impact the gastrointestinal tract? Just as observed in the serum, lower expression of TNF-α and IL-6 was detected in the colon. Fasting-induced adipose factor, a circulating lipoprotein lipase inhibitor, was significantly increased in the ileum compared to the SPI diet (1). This suggests that the GPs may aid in the suppression of fatty acid storage thereby attenuating the effects of diet-based metabolic syndrome. Further evidence in the ileum was provided by increased gene expression of proglucagon, a precursor of proteins associated with production of insulin and maintenance of gut barrier integrity (1). However, it would have been nice to see how these evidences compared with the controls used in this study as the data were not shown. Glut2, a gene for glucose transport, was also significantly lower in the jejunum tissue when compared to the mice on the SPI-diet (1).

Grape polyphenol-based diet led to decreased ratio of Firmicutes to Bacteroidetes and significant increase in the relative abundance of *Akkermansia muciniphila* in the cecal and fecal microbiota (1). This decrease in the proportion of Firmicutes to Bacteroidetes has been reported in other diet-induced obesity studies (2, 3). However, another study did not find a causal effect of Firmicutes to Bacteroidetes ratio in relation to obesity (4).

Similarly, the effects of dietary polyphenol from cranberry extract were evaluated in mice fed with high-fat/high-sucrose diet for 8 weeks (5). Cranberry extract prevented weight gain, enhanced insulin sensitivity, and reduced triglycerides in the jejunum. Cranberry extract-supplemented diet also led to a dramatic increase in *Akkermansia* (5). Furthermore, a reduction in body weight gain and insulin was observed in rats fed with a standard-chow diet supplemented with pterostilbene (6). Changes in the gut microbiota with increase in *Akkermansia* were also observed (6).

Of what importance is *Akkermansia* in the intestinal microbiota and how does it influence diet-induced obesity and metabolic disorders? *A. muciniphila* is a mucin degrading bacterium present in the mucus layer of the intestinal epithelium and may represent 3–5% of the gut microbiota in healthy adults (7). Several studies have shown an increase in *Akkermansia* in diet-induced obesity studies and correlates with the reduction of weight gain, adiposity, and improved glucose tolerance (1, 5, 8). Administration of live *A. muciniphila* reversed the symptoms of obesity and metabolic syndrome in HFD mice by reducing adiposity, inflammatory markers, insulin resistance, and improved gut barrier (7). Recently, it was shown that the introduction of capsaicin, a dietary polyphenol, led to an abundance of the genera *Akkermansia, Bacteroides*, and *Coprococcus* in mice fed with HFD and a decrease in weight gain (9). Potential mechanisms by which *Akkermansia* influences the host microbiota leading to these beneficial outcomes are depicted below (Figure 1).

**Figure 1:**
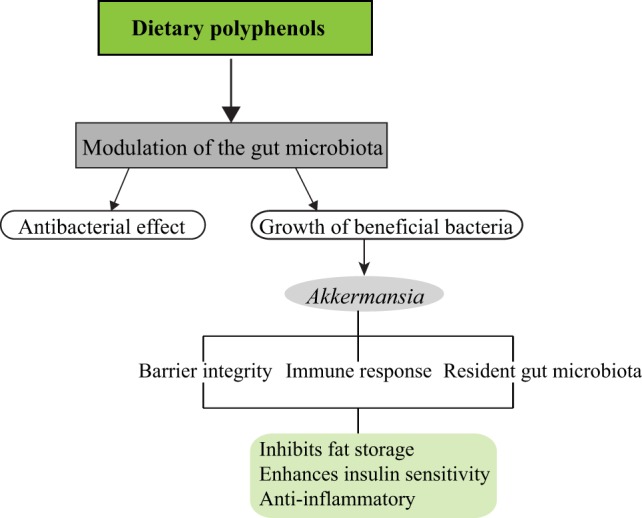
Dietary polyphenols and impact on the gut microbiota. Different classes of dietary polyphenols modulate the gut microbiota by various means. This could be by exerting antibacterial effect as observed in flavonoids on six pure cultures of intestinal bacteria (10) and stimulating the growth of beneficial bacteria in the gut microbiota such as *Akkermansia*. *Akkermansia*, in turn, generates short-chain fatty acids from the breakdown of mucins, which stimulates the goblet cells to produce more mucus thereby preserving/replenishing the intestinal barrier integrity. Mucus secretion is associated with the activation of the immune system, by preventing increased interaction of microbe-associated molecular patterns with intestinal epithelial cells, and stimulating other immune responses. This helps to reduce intestinal inflammation. *Akkermansia* may also influence resident gut bacteria by acting as an oxygen scavenger thereby, creating a favorable environment for the growth of strict anaerobes, which could have a synergistic effect on the host.

These and other studies suggest that dietary polyphenols play a role in the modulation of the gut microbiota that may favor positive outcomes. Understanding the mechanism of action of dietary polyphenols is likely to be key in the development of new diet-based therapies. This is because two different polyphenols can give complementary and dissimilar effects on the gut microbiota as observed in black tea and red wine grape extracts (11). As such, more studies are needed to unravel the bioactivity of this class of xenobiotics to fully understand their effects on the host.

## Author Contributions

The author confirms being the sole contributor of this work and approved it for publication.

## Conflict of Interest Statement

The author declares that the research was conducted in the absence of any commercial or financial relationships that could be construed as a potential conflict of interest.
